# Chronic treatment with *N*‐acetylcysteine decreases extinction responding and reduces cue‐induced nicotine‐seeking

**DOI:** 10.14814/phy2.13958

**Published:** 2019-01-10

**Authors:** Gregory L. Powell, Jonna M. Leyrer‐Jackson, Julianna Goenaga, Mark D. Namba, Jose Piña, Sade Spencer, Neringa Stankeviciute, Danielle Schwartz, Nicholas P. Allen, Armani P. Del Franco, Erin A. McClure, Michael Foster Olive, Cassandra D. Gipson

**Affiliations:** ^1^ Department of Psychology Arizona State University Tempe Arizona; ^2^ School of Life Sciences Arizona State University Tempe Arizona; ^3^ Department of Neuroscience Medical University of South Carolina Charleston SC; ^4^ School of Dental Medicine Lake Erie College of Osteopathic Medicine Bradenton Florida; ^5^ Department of Neuroscience University of Minnesota Minneapolis Minnesota; ^6^ Department of Psychiatry Medical University of South Carolina Charleston South Carolina

**Keywords:** N‐acetylcysteine, Nicotine, Relapse, Synaptic Plasticity

## Abstract

*N*‐acetylcysteine (NAC), a promising glutamatergic therapeutic agent, has shown some clinical efficacy in reducing nicotine use in humans and has been shown to reverse drug‐induced changes in glutamatergic neurophysiology. In rats, nicotine‐seeking behavior is associated with alterations in glutamatergic plasticity within the nucleus accumbens core (NAcore). Specifically, cue‐induced nicotine‐seeking is associated with rapid, transient synaptic plasticity (t‐SP) in glutamatergic synapses on NAcore medium spiny neurons. The goal of the present study was to determine if NAC reduces nicotine‐seeking behavior and reverses reinstatement‐associated NAcore glutamatergic alterations. Rats were extinguished from nicotine self‐administration, followed by subchronic NAC administration (0 or 100 mg/kg/d) for 4 days prior to cue‐induced reinstatement. NAcore synaptic potentiation was measured via dendritic spine morphology and mRNA and protein of relevant glutamatergic genes were quantified. Nicotine‐seeking behavior was not reduced by subchronic NAC treatment. Also, NAcore transcript and protein expression of multiple glutamatergic genes, as well as spine morphological measures, were unaffected by subchronic NAC. Finally, chronic NAC treatment (15 days total) during extinction and prior to reinstatement significantly decreased extinction responding and reduced reinstatement of nicotine‐seeking compared to vehicle. Together, these results suggest that chronic NAC treatment is necessary for its therapeutic efficacy as a treatment strategy for nicotine addiction and relapse.

## Introduction

Studies of United States (US)‐based smokers suggest that 68% seek cessation, but only 29% report using medication in cessation attempts (Centers for Disease Control and Prevention (CDC), 2011). Developments in the treatment of tobacco use disorder include replacement therapies and drugs that nonspecifically target nicotinic acetylcholine receptors, such as varenicline (Chantix^®^) (Cahill et al. 2014). However, these drugs have checkered clinical success promoting long‐term smoking cessation and often associate with adverse side effects (Moore et al. 2011). Given the negative health impact of tobacco and the recent surge in popularity of nicotine‐containing e‐cigarettes, there is great need for new pharmacotherapies to reduce craving and relapse to nicotine‐containing products.

The cysteine prodrug and antioxidant *N*‐acetylcysteine (NAC) has been used clinically since 1963 as a Food and Drug Administration (FDA)‐approved treatment of acetaminophen overdose and also acts as a mucolytic in the treatment of chronic bronchitis (Grandjean et al. 2000). As well, NAC is a safe, well‐tolerated compound (Berk et al. 2013). Importantly, NAC has shown translational value in the treatment of addiction across various levels of analyses and different drugs of abuse. NAC has been investigated as a potential clinical treatment for cocaine, cannabis, methamphetamine, and tobacco use disorders, as well as gambling disorder (see review by Grant et al. 2010, 2014; McClure et al. 2014; Deepmala et al. 2015; Roberts‐Wolfe and Kalivas 2015; Powell et al., 2019). Additionally, NAC has shown some efficacy as an adjunct pharmacotherapy in reducing cigarette craving (McClure et al. 2015). Preclinically, acute and chronic NAC treatment decreases nicotine self‐administration and inhibits cue‐induced nicotine‐seeking at different unit doses (Ramirez‐Niño et al. 2013; Moro et al. 2018). Mechanistically, NAC presumably increases glutamate clearance from the synaptic and extrasynaptic space following chronic drug self‐administration and withdrawal through upregulation of the glial glutamate transporter (GLT‐1), which is dysregulated in addiction (van Huijstee and Mansvelder 2015; Scofield et al. 2016). Dysregulation of glutamatergic signaling within the NAcore arises from alterations in glutamate transporters located on adjacent glia, where downregulation of GLT‐1 and the catalytic subunit of system Xc‐ (xCT) occurs following chronic administration of many drugs of abuse, including nicotine (Baker et al. 2003; Knackstedt et al. 2009; Sari et al. 2009; Gipson et al. 2013b; Alhaddad et al. 2014). System Xc‐ exchanges extracellular cysteine for glutamate in a 1:1 stoichiometry and plays a key role in regulating glutathione and oxidative stress levels in cells (Lewerenz et al. 2013). For example, drugs of abuse such as cocaine have been shown to reduce expression of xCT and cause enduring alterations in cellular redox homeostasis, contributing to alterations in behavioral plasticity that are thought to underlie relapse (Uys et al. 2011). Due to Xc‐ downregulation, extracellular glutamate responsible for activation of presynaptic type 2/3 metabotropic glutamate receptors (mGluR2/3) is reduced (Xi et al. 2002), disinhibiting synaptic release of glutamate during cue‐ and drug‐induced reinstatement and driving drug‐seeking behavior (Moran 2005). Finally, loss of GLT‐1‐dependent glutamate clearance exacerbates the effects of enhanced glutamate release during reinstatement (Knackstedt et al. 2010), as GLT‐1 clears approximately 90% of synaptic extracellular glutamate (Haugeto et al. 1996). Thus, pharmacotherapeutic targeting of GLT‐1 could restore glutamate homeostasis and reduce nicotine‐seeking behavior.

Previous research has shown that reinstatement of nicotine‐seeking is associated with rapid, transient synaptic potentiation (t‐SP) of medium spiny neurons (MSNs) of the nucleus accumbens core (NAcore) within the first 15 min of contingent‐conditioned cue exposure (*T*(time) = 15 min). Specifically, dendritic spine heads increase in diameter and then return to pre‐reinstatement levels by *T* = 45 (Gipson et al. 2013b). Furthermore, withdrawal from nicotine self‐administration has been associated with increased expression of alpha‐amino‐3‐hydroxy‐5‐methyl‐3‐isoxazole propionic acid (AMPA) and *N*‐methyl‐D‐aspartate (NMDA) receptors as well as potentiated excitatory postsynaptic currents in MSNs within the NAcore. These results, as well as the positive correlations of AMPA/NMDA ratio and spine head diameter with active lever presses during a reinstatement session, suggest that postsynaptic transient potentiation may increase motivated motor output and thus drive relapse behavior. Targeting sites within the limbic motor subcircuit to reverse nicotine‐induced neurobiological alterations may reduce nicotine‐seeking and restore glutamatergic signaling. These targets potentially influence relapse vulnerability to multiple drugs of abuse that are known to cause similar maladaptations (Kalivas 2009; Shen et al. 2011; Gipson et al. 2013a,2013b; Mulholland et al. 2016), illustrating possible shared neural substrates that could be targeted in the treatment of addiction across drug classes.

To determine if NAC modifies neurobiological alterations associated with withdrawal from nicotine self‐administration (Gipson et al. 2013a,2013b), transcript and protein expression levels of glutamatergic genes, as well as t‐SP during cue‐induced reinstatement of nicotine‐seeking behavior following subchronic (5 days) NAC treatment were analyzed. Although NAC alters glutamatergic protein expression and subsequent drug‐seeking behavior in rodent cocaine self‐administration models (Sari et al. 2009; Knackstedt et al. 2010; Fischer et al. 2013; Reissner et al. 2015), the specific molecular mechanisms underlying these processes are largely unknown. Thus, mRNA and protein expression were examined within the NAcore to determine whether NAC alters key glutamatergic substrates within a nicotine self‐administration model. Additionally, as t‐SP has been observed following cue‐induced reinstatement of nicotine‐seeking behavior, we examined if NAC administration had the ability to block t‐SP following cue‐induced reinstatement of nicotine. Given that clinical studies showing NAC‐induced reductions in drug craving (see Gray et al. 2012; Grant et al. 2014; McClure et al. 2014) administered NAC to participants over the course of several weeks, we next examined the effects of chronic NAC treatment (15 days) on nicotine‐seeking behavior to determine if extended NAC treatment leads to successful reduction in nicotine‐seeking behavior.

## Materials and Methods

### Subjects and surgery

Male Sprague Dawley rats (*n* = 93; 225–250 g; Charles River) were housed on a 12‐h reverse light cycle and had ad libitum access to food and water prior to self‐administration procedures. All procedures were performed during the animals’ active phase. Animals were handled daily. All animal use practices were approved by Institutional Animal Care and Use Committees of Arizona State University and the Medical University of South Carolina.

### Self‐administration, extinction, and reinstatement procedures

Prior to surgical implantation of indwelling catheters, animals were placed on food restriction (20 g/day) and placed in operant chambers for 15 h to undergo food training. Jugular catheters (SILASTIC ^®^, Dow Corning, Midland, MI) were implanted under anesthesia with intramuscular ketamine HCl (87.5 mg/kg) and xylazine (5 mg/kg). The catheter was tunneled subcutaneously from a small incision between the shoulder blades and anchored into the jugular vein. The alternate end was then attached to a backpack‐style cannula (PlasticsOne^®^, Roanoke, VA). Animals were given 1‐week recovery time with daily cefazolin treatment (100 mg/kg, intravenous (IV)) for 7 days, as well as 3 days of subcutaneous meloxicam injections (1 mg/ml). Animals were given daily infusions of heparinized saline (100 usp/mL, IV) to promote patency of catheters throughout the duration of the self‐administration timeline.

Following the week of postoperative care, animals were placed into nicotine self‐administration (MP Biomedicals, Solon, OH dissolved in 0.9% sterile saline and pH adjusted to 7.2; the final concentration was 0.2 mg/mL) on a fixed ratio 1 (FR1) schedule of reinforcement. Nicotine was delivered across a 5.9‐s infusion duration after a lever press on a designated active lever. During the infusion, lights above the levers illuminated and a tone (2900 Hz) was presented, immediately followed by a 20‐ sec timeout period when any additional presses on the active lever were recorded but had no consequences (Gipson et al. 2011, 2013b). An inactive lever was extended at all times and presses were recorded, but produced no consequences. Sessions lasted 2 h, with the first three sessions capped at 30 infusions to prevent aversion to nicotine. Animals that achieved at least 10 sessions with at least 10 infusions and an active‐to‐inactive lever press ratio of at least 2:1 were moved into extinction training (*n* = 87; 93.5%).

Extinction training consisted of 2 h sessions with active and inactive levers present, but neither lever produced conditioned stimuli or infusions. For subchronic NAC treatment, animals received a minimum of 10 extinction sessions prior to beginning daily intraperitoneal injections of NAC (Sigma‐Aldrich: catalog #A7250; dissolved in 0.675 M NaOH in saline and then adjusted to pH 7.3–7.4). Injections occurred 2 h prior to operant sessions as previously published (Moussawi et al. 2009), and were administered on the last 4 days of extinction as well as the fifth injection day (2 h prior to reinstatement) for subchronic treatment. During reinstatement sessions, cues were presented upon presses of the active lever, however, no nicotine was available for either 2 h (*T* = 120) or 15 min (*T* = 15). Animals were immediately sacrificed upon completion of the reinstatement session. A nonreinstating group (*T* = 0) was sacrificed 2 h after the final NAC injection without exposure to the operant environment for spine morphological assessment.

Two cohorts of animals utilized for quantitative reverse transcription polymerase chain reaction (RT‐q PCR) analysis received timed saline infusions in place of self‐administered nicotine. Animals were given 10 sessions of saline administration before entering the extinction phase. Levers were present in operant chambers as before, but pressing on both levers produced no consequences. Saline administrations (13 total) were accompanied by the same light and tone stimuli as in nicotine self‐administration. Animals were given 14 extinction sessions, with NAC (100 mg/kg) or vehicle injections administered on the last 4 days. For animals tested for transcript expression with RT‐qPCR, prior to the next session, a fifth NAC injection was given and animals returned to the timed saline infusion environment 2 h later. Upon completion of the 2 h session, animals were deeply anesthetized with isoflurane and sacrificed. For animals tested for protein expression of GLT‐1 using Western blotting, no reinstatement session was given (*T* = 0), thus NAC or vehicle injections were adjusted to extinction days 10–14, and no treatment was given on the day of sacrifice.

To determine if chronic administration of NAC alters nicotine‐seeking behavior, an additional cohort of animals (*N* = 18) received NAC injections (0 or 100 mg/kg, IP) prior to each extinction session as well as prior to a 2‐h reinstatement session.

### Dendritic spine morphology

Dendritic spines of NAcore MSNs (Fig. 4B, C) were imaged using a confocal microscope (Leica SP5 (Arizona State University) or Zeiss LSM510 (Medical University of South Carolina)) after cell impregnation with DiI. For both confocal microscopes, DiI was excited using the helium/neon 543 nm laser line. Acquisition of DiI‐labeled dendrite images was made via optical sectioning using a 63× oil immersion objective (HCX PL APO CS 63x/1.4‐0.6NA). On the Leica microscope, image resolution was set to 512 × 512 pixels with a pixel scale of 0.15 × 0.15 *μ*m. Each dendrite was scanned at 0.21 *μ*m intervals along the z‐axis and reconstructed in three dimensions for later analysis. On the Zeiss microscope, images were acquired via optical sectioning at 63x magnification with an oil immersion objective (Plan‐Apochromat, Zeiss; N.A. = 1.4, working distance=90 *μ*m). Pixel scale was set to 0.07 × 0.07 *μ*m with *z*‐axis steps at 0.1 *μ*m. All images were deconvolved using Autoquant (Media Cybernetics), followed by 3D‐rendering using the Surpass module in the Imaris software package (Bitplane). Spine measurements were made a minimum of 50 *μ*m from the soma and dendritic length for each measurement was between 45 and 55 *μ*m. Minimum spine head diameter was set at ≥0.143 *μ*m, and spine neck length was measured from the shaft of the dendrite to the spine head.

### mRNA quantification using RT‐qPCR

At *T* = 120, animals were anesthetized using isoflurane and quickly decapitated. Brain tissue was removed and flash frozen in methylbutane before storage at −80°C. Tissue slices of the NAcore were made at 2 mm thick and NAcore tissue was isolated using a 1.25‐mm biopsy punch (Harris Unicore™). mRNA from NAcore tissue was extracted via standard Trizol^®^ (Invitrogen) extraction procedure, followed by precipitation of RNA in ethanol and glycogen. cDNA was prepared using 15 ng of purified mRNA using the SuperScript II First‐Strand Synthesis system (Life Technologies), then quantified using Power SYBR Green Master Mix (Life Technologies) with primers designed against the following targets: *Slc1a2* (Forward: CAGAGAGGCTGCCCGTTAAA; Reverse: CTTCCACCTGCTTGGGCATA), *Grin2a*(Forward: CATGGCTGACAAGGATCCGA; Reverse: TATCCCAGCCCACAAAGCTG), *Grin2b*(Forward: AACCAAGAGAGCCGACTAGC; Reverse: ACACCAACCAGAACTTGGGG), *Gria1* (Forward: GGACAACTCAAGCGTCCAGA; Reverse: CACAGTAGCCCTCATAGCGG), and *Gria2* (Forward: GCATCGCCACACCTAAAGGA; Reverse: GGGCACTGGTCTTTTCCTTGG), with *Gapdh* as a control transcript(Forward: CCACAGTCCATGCCATCACT; Reverse: GCCTGCTTCACCACCTTCTTG) (Eurofins Genomics, Louisville, KY). Relative expression was calculated using the comparative 2^−ΔCt^ methodology (Schmittgen and Livak 2008), where expression of transcripts of interest are compared against the control transcript, *Gapdh*. Each sample plate included a no reverse transcriptase reaction, with zero no‐reverse transcriptase controls amplified.

### Protein quantification using western blots

Following reinstatement testing, rats were rapidly decapitated and NAcore tissue was dissected over ice. Tissue was homogenized in 200 *μ*L of ice‐cold radioimmunoprecipitation assay (RIPA) lysis buffer containing 1:100 protease and phosphatase inhibitors. Homogenates were centrifuged at 10,000*g* for 5 min at 4°C and the supernatant was collected and stored at −80°C. Protein concentrations were determined by the BCA method (Thermo Scientific). Samples were prepared in 4x lithium dodecyl sulfate (LDS) sample buffer (Invitrogen) and incubated for 10 min at 70°C. Equal microgram quantities of protein were loaded onto a 4–12% Bis–Tris gel (Invitrogen) and transferred to a nitrocellulose membrane using semidry transfer conditions for 6 min (iBlot, Invitrogen). Membranes were blocked for 1.5 h in tris‐buffered saline plus 0.1% Tween‐20 (TBST) containing 5% nonfat milk and incubated overnight at 4°C in primary antibody. Membranes were washed 3 × 10 min in blocking buffer, incubated in secondary antibody for 2 h at room temperature (Table 1), and washed 3 × for 10 min in TBST. Enhanced chemiluminescence substrate (Thermo Scientific) was used to detect protein expression on x‐ray film, and band density was quantified using NIH ImageJ software. Protein expression levels were normalized to glyceraldehyde‐3‐phosphate dehydrogenase (GAPDH).

**Table 1:** Antibodies used in Figure 2F

Target	Catalog #	Lot #	Primary Antibody Dilution	Secondary Antibody Dilution
GLT‐1	Abcam ab41621	GR30201 4‐1	1:1000	1:25,000
GluN2A	Abcam ab169873	GR283444‐6	1:1000	1:10,000
GluN2B	Abcam ab65783	GR242023‐1	1:1000	1:10,000
GluA1	Abcam ab31232	GR259313‐1	1:1000	1:10,000
GAPDH	Cell‐Signaling Technology D16H11	6	1:1000	1:5,000
Goat Anti‐Rabbit (HRP)	Abcam ab97080	GR258796‐3	N/A	1:5000–1:25,000 (see above)

### Data analysis

Behavioral and spine data were analyzed using analysis of variance (ANOVAs) with Bonferroni‐corrected *t* tests post hoc, where appropriate. Analysis of behavioral data only included animals that achieved self‐administration criteria. A total of five animals across all experiments were removed as outliers during reinstatement testing, as their lever pressing during the reinstatement session was greater than two standard deviations above or below the mean (Ahmed et al. 2000; Pockros‐Burgess et al. 2014; Weiland et al. 2015). Behavior analysis during self‐administration, extinction, and reinstatement was performed using two‐way, mixed measures ANOVA with treatment as a main factor and session (extinction vs. reinstatement, where applicable) as a repeated‐measure factor. Cumulative cue presentations during reinstatement were analyzed using linear regression analysis. Spine morphological measures were averaged across all spines from 1 to 5 dendritic segments from all cells (one segment per cell) by rat. Spine morphology data were then analyzed by two‐tailed *t* tests between animal means. Cumulative frequency distributions were analyzed after using a two‐way ANOVA with diameter or neck length and treatment as main factors. mRNA and protein expression were analyzed using two‐tailed *t* tests unless otherwise indicated. Statistical tests were performed in Graphpad Prism 8.0 or SPSS 25 (IBM) software packages, and *P* < 0.05 was considered statistically significant.

## Results

### Subchronic NAC treatment does not decrease nicotine‐seeking behavior

**Figure 1:**
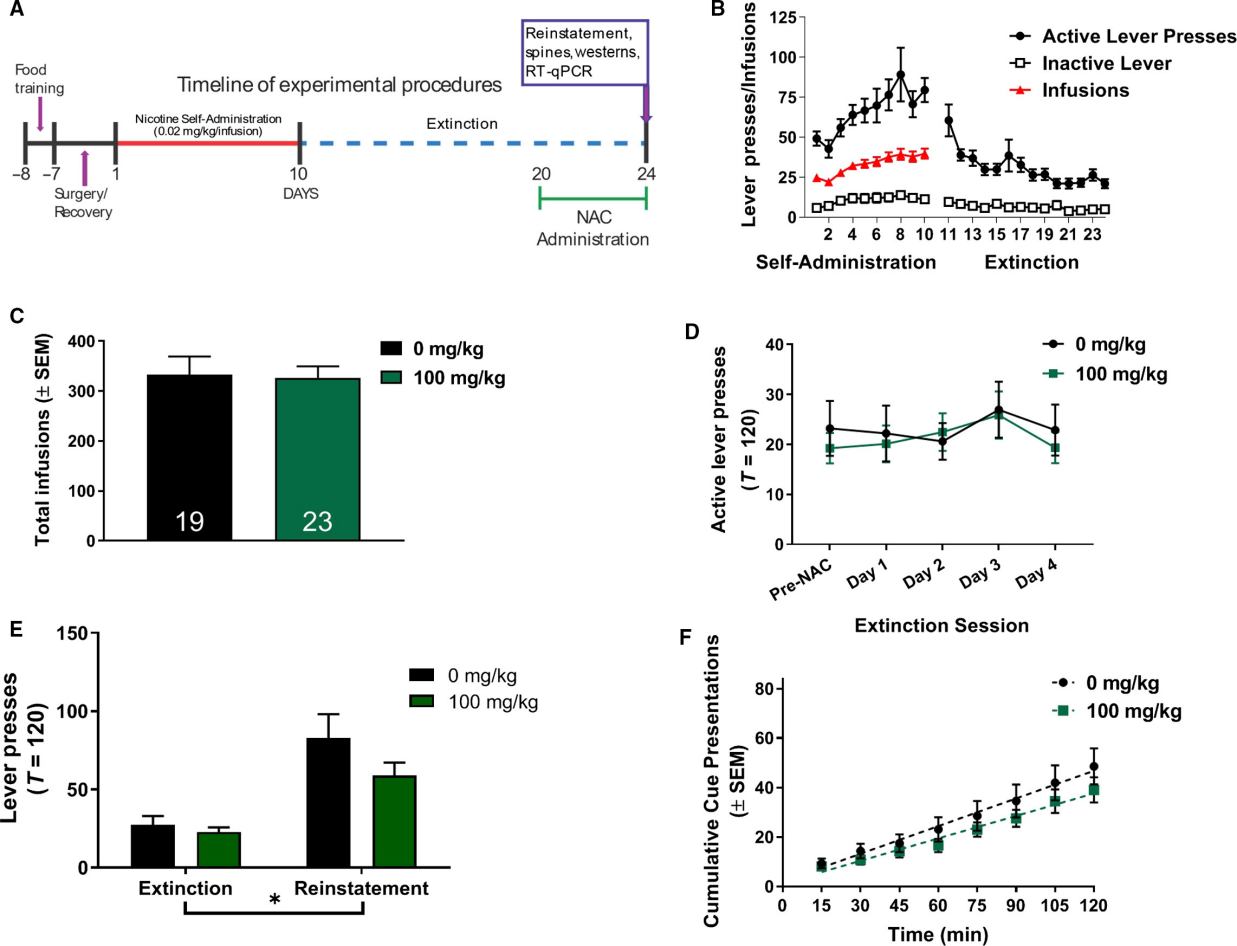
Subchronic N‐acetylcysteine (NAC) does not attenuate nicotine‐seeking behavior. (A) A timeline of experimental procedures including nicotine self‐administration, extinction training, reinstatement of nicotine‐seeking, NAC administration, and tissue collection (to be used for either RT‐qPCR, western blots, or dendritic spine morphology). (B) Rats acquired lever pressing to the active lever to receive intravenous infusions of nicotine (0.02 mg/kg/infusion) paired with a light+tone compound stimulus during self‐administration. During extinction training, active lever pressing decreased to inactive lever press rates due to no programmed consequence. (C) No difference was found for the total number of infusions received throughout self‐administration, prior to NAC or vehicle treatment. (D) Administration of NAC during extinction had no significant effect on active lever pressing. (E) In a 2‐h cue‐reinstatement session, animals receiving 0 or 100 mg/kg NAC showed no significant effects of treatment on mean active lever pressing. (F) Upon examination of the time‐course of active lever pressing during the 2‐h reinstatement session, NAC treatment did not significantly reduce cumulative active lever pressing compared to vehicle‐treated animals. *P < 0.0001, significant main effect of session (extinction vs. reinstatement). The bar in (E) indicates a significant main effect of session.

There were no significant differences in total nicotine infusions between treatment groups randomized to subchronic NAC treatment or vehicle (Fig. 1C; *t*_40_ = 0.1511 = 0.653, *P *>* *0.05). Subchronic NAC treatment did not significantly alter active lever pressing during extinction (Fig. 1D; no main effect of treatment: *F*_1,40_ = 0.13, *P *>* *0.05, session: *F*_3.28,131.3_ = 1.18, *P *>* *0.05, or interaction: *F*_4,160_ = 0.30, *P *>* *0.05). Additionally, a two‐way ANOVA was conducted on cued reinstatement data (Fig. 1E). A significant main effect of session was found (*F*_1,40_ = 40.73, *P *<* *0.0001), indicating that rats reinstated to nicotine‐conditioned cues. However, no significant main effect of treatment was observed (*F*_1,40_ = 1.94, *P *>* *0.05), nor a treatment × session interaction. In addition, linear regression analysis of cumulative cue presentations during the 2 h reinstatement session (Fig. 1F) indicated no significant effect of treatment on the slope (*F*_1,340_ = 1.26, *P *>* *0.05), indicating no difference in the rate reinstatement due to NAC administration.

### The effects of subchronic NAC treatment on glutamatergic transcript and protein expression

**Figure 2:**
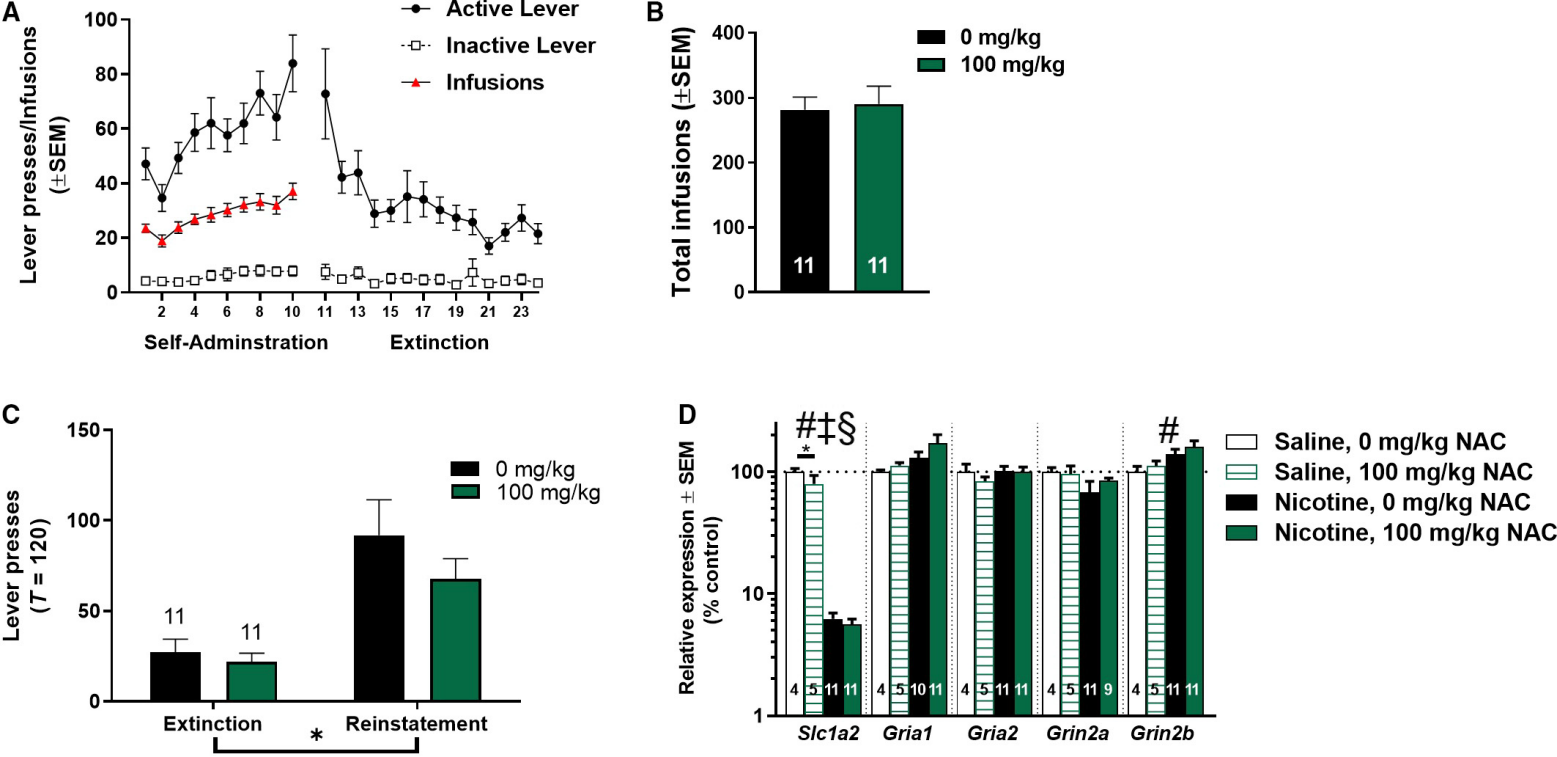
The effect of subchronic N‐acetylcysteine (NAC) on glutamatergic genes. (A) Lever presses and infusions during nicotine self‐administration and extinction training of rats tested for transcript expression following administration of 0 or 100 mg/kg NAC prior to the last four sessions of extinction and the reinstatement session. (B) No difference was found for the total number of infusions received throughout self‐administration, prior to NAC or vehicle treatment. (C) High‐dose NAC did not significantly reduce active lever pressing during a 2‐h reinstatement test prior to sacrifice for gene expression analysis. (D) Glutamatergic transcripts of interest (*Slc1a2*,* Gria1*,* Gria2*,* Grin2a*, and *Grin2b*) relative to the endogenous control gene GAPDH revealed no differences in mRNA expression due to subchronic 100 mg/kg NAC treatment. Expression is shown as a percentage of control animals. N per group for each gene is listed with each bar. **P < 0.0001 main effect of session (extinction vs. reinstatement). #*P* < 0.05 main effect of drug (saline vs. nicotine). ‡*P* < 0.05 main effect of treatment (0 vs. 100 mg/kg NAC). §*P* < 0.05 interaction between drug and treatment. *P < 0.05 significant difference between 0 and 100 mg/kg NAC in saline animals. The bar in **(C)** indicates a significant main effect of session.

For PCR experiments, no significant differences were found in total infusions during self‐administration (Fig. 2B; *t*_20_ = 0.27, *P *>* *0.05). Using a two‐way ANOVA to examine active lever pressing during cue reinstatement demonstrated significant main effects of session (*F*_1,20_ = 26.79, *P *<* *0.0001) were observed, but no main effect of treatment was seen (*F*_1,20_ = 1.21, *P *>* *0.05). No significant interactions were observed. Among all transcripts tested (Fig. 2D), a main effect of drug (nicotine vs. saline) was only observed for *Slc1a2* (*F*_1,27_ = 278.7, *P *<* *0.0001) and *Grin2b* (*F*_1,27_ = 4.251, *P *<* *0.05). Furthermore, a main effect of treatment (0 vs. 100 mg/kg NAC) was found on *Slc1a2* expression (*F*_1,27_ = 4.537, *P *<* *0.05), but for no other transcripts tested. These results indicate that NAC decreased *Slc1a2* expression in a system unexposed to nicotine. No significant interaction effects were found with any transcript tested.

**Figure 3:**
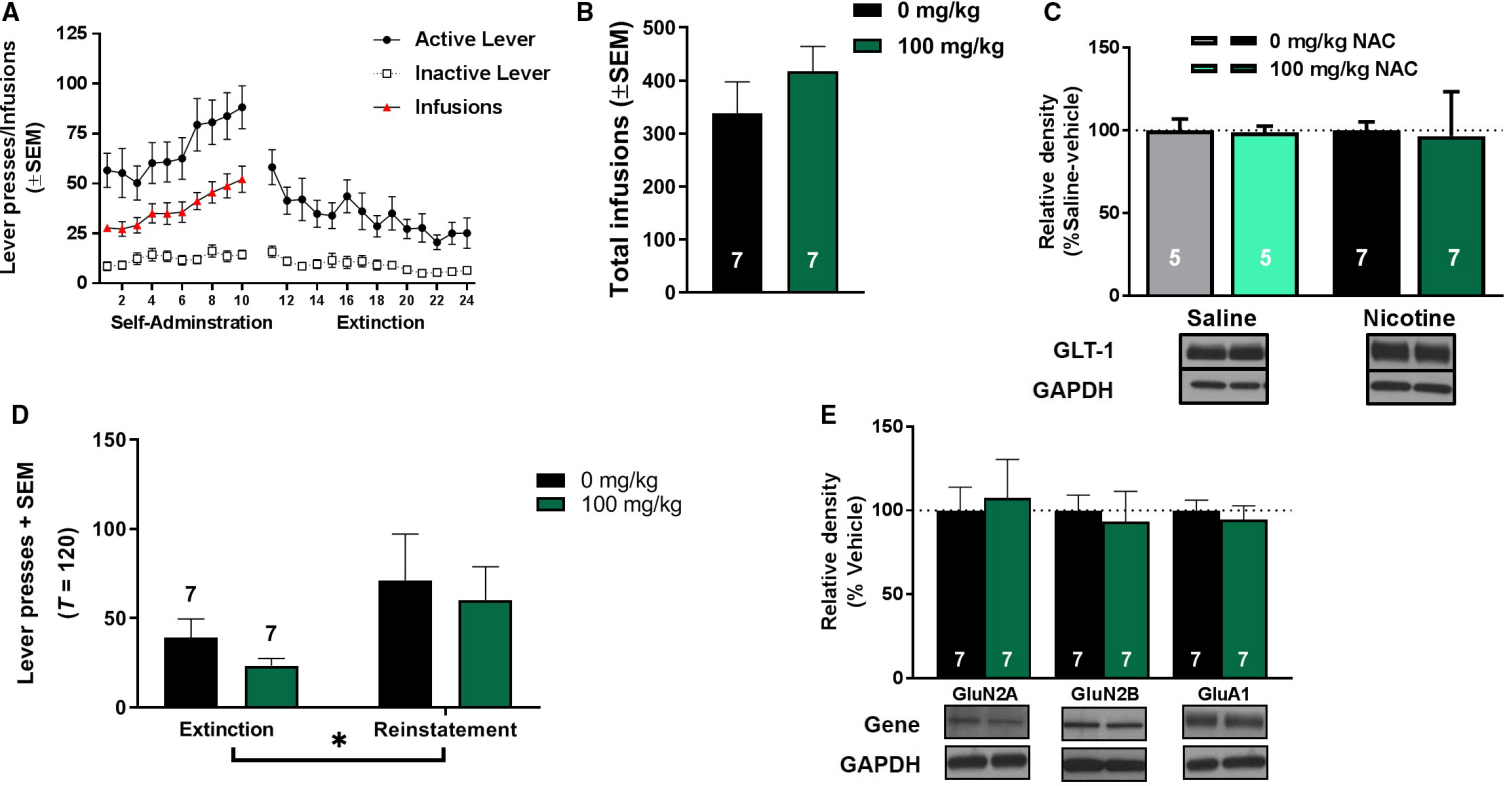
Subchronic *N*‐acetylcysteine (NAC) does not alter nucleus accumbens core (NAcore) protein expression. (A) Lever presses and infusions during nicotine self‐administration and extinction training for animals tested for protein expression following treatment with 0 or 100 mg/kg NAC. (B) No difference was found for the total number of infusions received throughout self‐administration, prior to NAC or vehicle treatment. (C) Expression of GLT‐1 protein was not significantly different between groups of nicotine self‐administering and timed saline infusions, as well as between vehicle and 100 mg/kg NAC. Expression is shown as a percentage of animals receiving timed saline infusions and vehicle treatment (0 mg/kg NAC), and calculated using GAPDH as a control protein. (D) NAC did not significantly reduce active lever pressing during a 2‐h reinstatement test prior to sacrifice for protein expression analysis. (E) Treatment with NAC did not significantly alter levels of glutamatergic proteins of interest. Expression is shown as percentage of control animals, and calculated using GAPDH as a control. *P < 0.05 main effect of session (extinction vs. reinstatement). The bar in (D) indicates a significant main effect of session.

For western blot experiments, no effects of NAC administration were found in a cohort tested for changes in protein expression (Fig. 3A). No difference in total infusions during self‐administration was observed between groups later administered 0 or 100 mg/kg NAC (Fig. 3B; *t*_12_ = −1.05, *P *>* *0.05). To determine if NAC altered expression of the glutamate transporter GLT‐1 as has been reported previously (Sari et al. 2009; Knackstedt et al. 2010; Fischer et al. 2013; Reissner et al. 2015), we examined protein concentrations in animals receiving nicotine self‐administration as well as timed saline infusions, with vehicle or NAC treatment during extinction (Fig. 3C). There were no significant main effects on GLT‐1 expression due to drug (nicotine vs. saline; *F*_1,20_ = 0.0038; *P *>* *0.05) or treatment (vehicle vs. NAC; *F*_1,20_ = 0.021, *P *>* *0.05), nor any interaction between drug and treatment (*F*_1,20_ = 0.0038, *P *>* *0.05). The effects of NAC on reinstatement within the protein analysis cohort were analyzed using a three‐way ANOVA, with session as a within‐subjects factor, treatment as a between‐subjects factor, and lever as a within‐sessions factor (Fig. 3D). Significant main effects were observed for session (*F*_1,12_ = 6.595, *P *<* *0.05) and lever (*F*_1,12_ = 16.794, *P *<* *0.001), but no effects of treatment were found (*F*_1,12_ = 0.507, *P *>* *0.05). A significant session × lever interaction was observed (*F*_1,12_ = 8.511, *P *<* *0.05), but no other interactions reached significance. NAC had no significant effects on expression of glutamatergic proteins (Fig. 3E), specifically GLT‐1 (*t*_12_ = 0.126, *P *>* *0.05), GluN2A (*t*_12_ = −0.282, *P *>* *0.05), GluN2B (*t*_12_ = 0.330, *P *>* *0.05), and GluA1 (*t*_12_ = 0.531, *P *>* *0.05). NAC administration had no significant effect on expression of the control protein, GAPDH.

### Cue‐induced nicotine‐seeking does not induce transient synaptic potentiation

**Figure 4:**
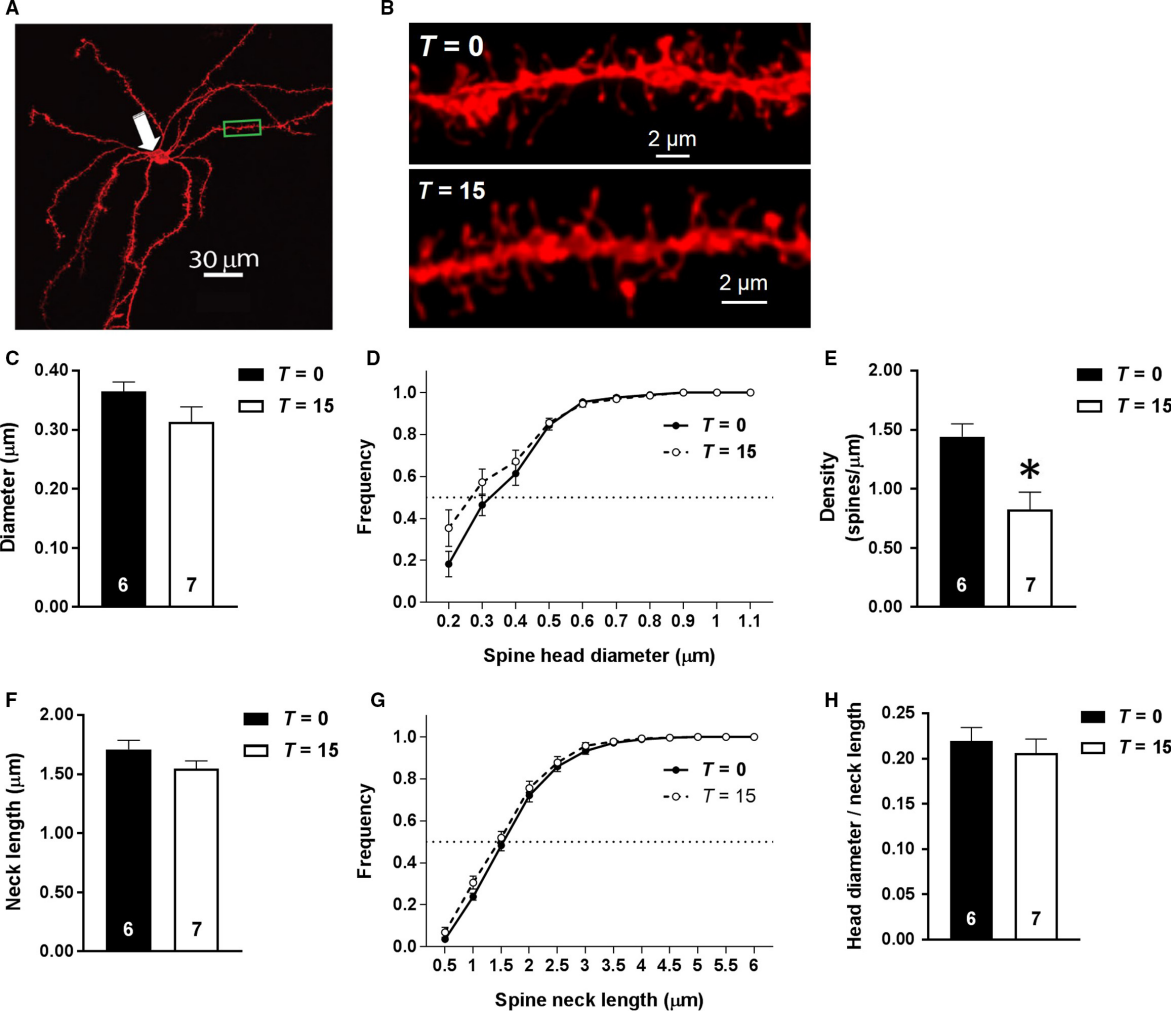
Effects of cue‐induced nicotine‐seeking on dendritic spine morphology. (A) A representative medium spiny neuron (MSN) with soma denoted by a white arrow and a sample dendrite used for analysis highlighted within a green box. (B) Sample dendritic segments of MSNs from animals at *T* = 0 **(** *top* **)** or *T* = 15 **(** *bottom* **)**. The comparison of mean spine head diameter of animals at *T* = 0 and *T* = 15, as well as the cumulative frequency distribution of spine head diameters are shown in (C) and (D), respectively. Additionally the comparison of spine head density (E), spine neck length (F), frequency distribution of spine neck length, (G) and the ratio for spine head diameter to spine neck length (H) between animals undergoing a 15‐min reinstatement session (*T* = 15) and those that did not (*T* = 0) are shown. *P < 0.05 compared to animals at T = 0.

Dendritic spine morphological features were compared (see representative micrographs in Fig. 4A and B) between nicotine‐extinguished animals that underwent reinstatement (*T* = 15; *n* = 7) and those that did not (*T* = 0; *n* = 6). Reinstatement of nicotine‐seeking did not alter mean spine head diameter (*t*_11_ = 1.632, *P *>* *0.05; Fig. 4C), mean spine neck length (*t*_11_ = 1.648, *P *>* *0.05; Fig. 4F), or ratio of spine head diameter to neck length (*t*_11_ = 0.612, *P *>* *0.05; Fig. 4H). As well, reinstatement of nicotine‐seeking significantly decreased spine density (*t*_11_ = 3.277; *P *<* *0.01; Fig. 4E). To determine if the distributions of spine head diameters and neck lengths were altered by reinstatement of nicotine‐seeking, we compared the cumulative frequency distributions with a two‐way ANOVA with reinstatement and bin size as main effects. Reinstatement of nicotine‐seeking did not alter the frequency distribution of spine head diameter (*F*_1,12_ = 1.176, *P *>* *0.05; Fig. 4D) or spine neck length (*F*_1,12_ = 1.487, *P *>* *0.05; Fig. 4G). These results indicate that initiated reinstatement did not induce rapid, transient changes in spine morphology as previously found (Gipson et al. 2013a,2013b).

### Subchronic NAC treatment does not alter spine morphology

**Figure 5:**
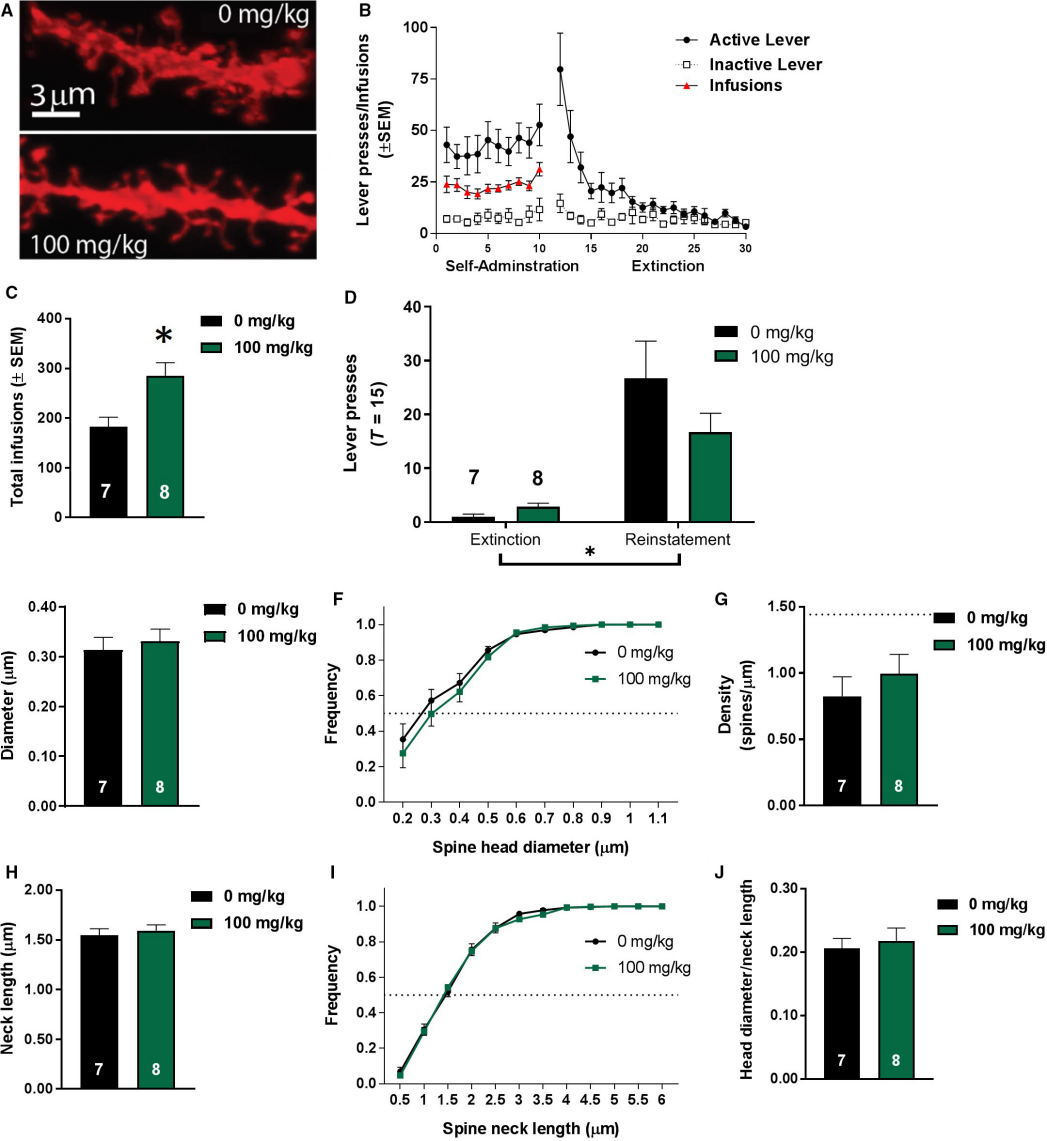
Subchronic *N*‐acetylcysteine (NAC) does not inhibit nicotine reinstatement or t‐SP. (A) Representative micrographs of dendritic spines from animals receiving 0 **(** *top* **)** or 100 mg/kg NAC **(** *bottom* **)**. (B) Self‐administration and extinction of rats receiving either 0 or 100 mg/kg NAC prior to sacrifice for dendritic spine morphology analysis. (C) Total infusions received by group throughout self‐administration. (D) Treatment with 100 mg/kg NAC did not significantly reduce active lever pressing during a 15 min reinstatement session compared to vehicle‐treated animals. (E) High‐dose NAC (100 mg/kg) did not significantly alter mean spine head diameter. No effects of high‐dose NAC (100 mg/kg) were observed on the (F) cumulative frequency distribution of spine neck diameter or spine neck length (I) at *T* = 15. Comparisons of spine density, mean spine neck length, and head diameter to neck length ratio are shown in (G), (H), and (J), respectively. Dashed line in (G) represents basal (*T* = 0, vehicle‐treated) levels. *P <* *0.05 compared to animals receiving 0 mg/kg NAC in (C). *P < 0.0001 main effect of session (extinction vs. reinstatement) in (D).

Next, spine morphological measures were analyzed at *T* = 15 following subchronic NAC treatment. Representative micrographs of dendritic spines are shown in Figure 5A, and self‐administration, extinction, total infusions, and reinstatement results for this cohort of animals are illustrated in Figure 5B, C, and D, respectively. Analyses indicated a significant increase in infusions during self‐administration training for animals that received 100 mg/kg NAC (main effect of dose: *t*_13_ = 3.114, *P *<* *0.01; Fig. 5C). Reinstatement testing during a 15‐min session (Fig. 5D) revealed a main effect of session (extinction vs. reinstatement, *F*_1,24_ = 25.89, *P *<* *0.0001), but no main effect of treatment (*F*_1,24_ = 1.096, *P *>* *0.05), as well as no significant interaction between session and treatment (*F*_1,24_ = 2.325, *P *>* *0.05). Comparison of mean morphological parameters revealed no significant differences in spine head diameter (*t*_13_ = −0.512, *P *>* *0.05; Fig. 5E), spine density (*t*_13_ = −0.809, *P *>* *0.05; Fig. 5G), spine neck length (*t*_13_ = −0.545, *P *>* *0.05; Fig. 5H), or head diameter to neck length ratio (*t*_13_ = −0.442, *P *>* *0.05; Fig. 5J). To determine if NAC was able to alter spine density to *T* = 0 vehicle‐treated levels, a one‐way ANOVA comparing three groups (*T* = 0 vehicle, *T* = 15 vehicle, and *T* = 15 100 mg/kg NAC) indicated a significant effect of group (*F*_2,18_ = 4.714, *P < *0.05). However, Bonferroni‐adjusted post hoc comparisons indicated only a significant difference between *T* = 0 vehicle‐treated and *T* = 15 vehicle‐treated spine density (*t*_18_ = 2.991, *P < *0.05). Comparison of the total number of infusions acquired during self‐administration and mean spine head diameter or mean spine density yielded no significant correlations (spine head diameter: *F*_1,13_ = 0.40, *P *>* *0.05; *r* ^2^ = 0.03; spine density: *F*_1,13_ = 1.668, *P* >* *0.05; *r* ^2^ = 0.11). Additionally, we compared the frequency distributions for spine head diameter and neck length using a two‐way ANOVA with treatment and bin as main factors. No difference in cumulative frequency distribution of spine head diameters was observed (main effect of treatment: *F*_1,13_ = 0.414, *P* >* *0.05; Fig. 5F). Additionally, treatment with 100 mg/kg NAC prior to a 15‐min reinstatement session did not alter the cumulative frequency distribution of spine neck lengths (*F*_1,13_ = 0.186, *P* >* *0.05; Fig. 5I).

### Chronic NAC treatment decreases extinction responding and nicotine‐seeking behavior

**Figure 6:**
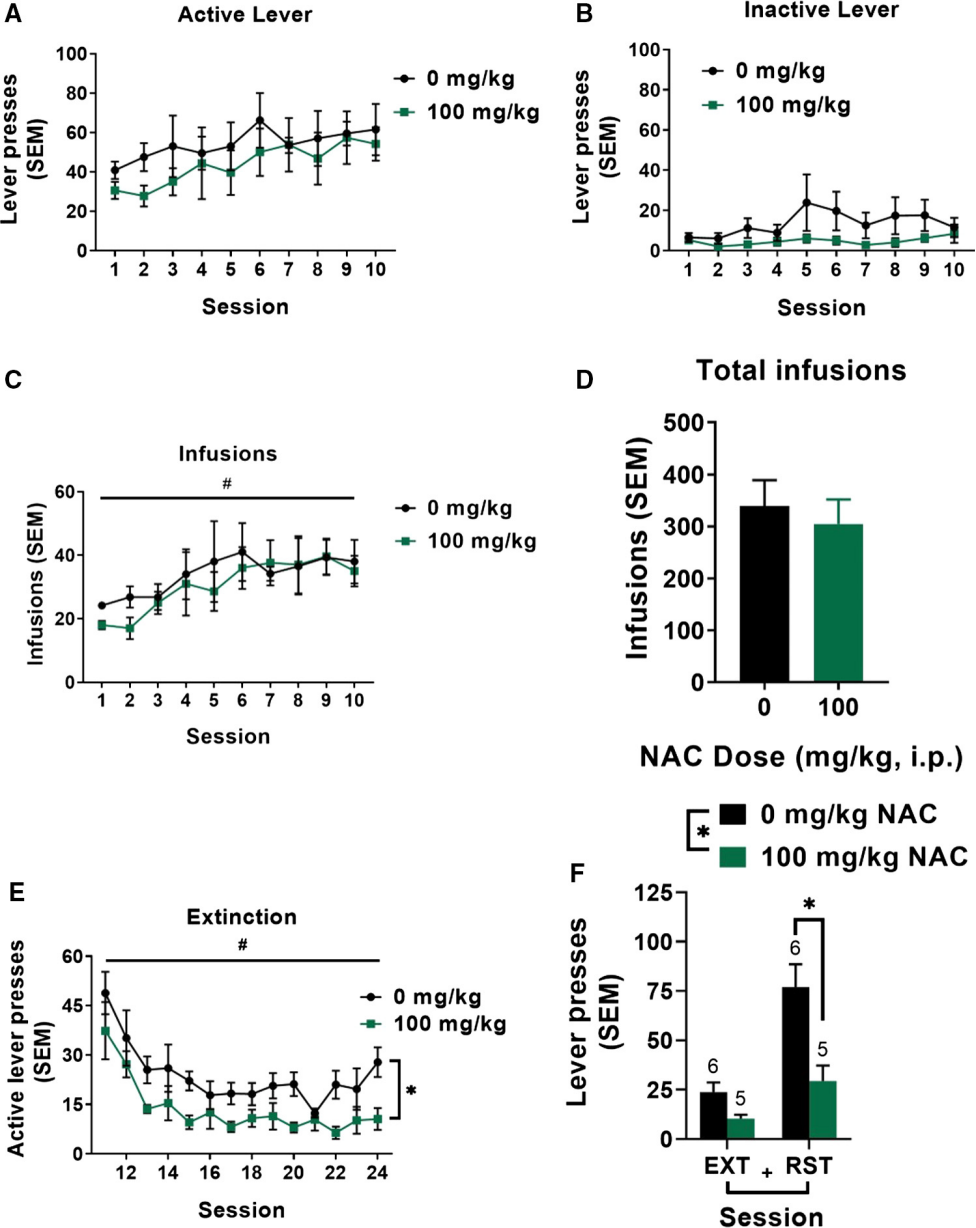
Chronic *N*‐acetylcysteine (NAC) attenuates nicotine‐seeking behavior. Active (A) and inactive lever presses (B) during self‐administration training for animals treated with 0 or 100 mg/kg NAC. No differences were detected due to treatment or session. (C) The number of infusions earned by group during nicotine self‐administration. No significant differences were detected due to treatment, however, a significant effect of session was observed indicating all animals increased infusions earned as training progressed. (D) The total number of infusions earned by treatment group collapsed across session. No significant differences were detected. (E) Active lever pressing during 2‐h extinction sessions significantly decreased across sessions, and was significantly reduced with chronic NAC treatment. (F) Following extinction, rats significantly reinstated to nicotine‐conditioned cues during a 2‐h reinstatement test. Additionally, NAC treatment significantly reduced active lever pressing regardless of session. Bonferroni‐corrected comparisons of vehicle versus treatment lever pressing indicated 100 mg/kg NAC significantly decreased active lever pressing during reinstatement. *P < 0.05, significant main effect of treatment. #*P* < 0.05, significant main effect of session. +*P* < 0.05, significant main effect of session.

In an additional cohort of rats, no significant main effects of treatment, session, or interactions between treatment and session were observed for active (Fig. 6A) or inactive (Fig. 6B) lever pressing during self‐administration training. A significant main effect of session (*F*_9,81_ = 3.76, *P* <* *0.001) was found on the number of infusions earned during self‐administration training (Fig. 6C), but no main effect of treatment or interaction between session and treatment was observed. Furthermore, no differences in the total number of infusions earned prior to NAC treatment were observed among groups (Fig. 6D; *t*_9_ = 0.49, *P *>* *0.05). During extinction training (Fig. 6E), a significant main effect of session (*F*_4.64, 41.79_ = 8.21, *P *<* *0.001) was found, indicating a significant reduction of active lever pressing with extinction training. Additionally, a significant main effect of treatment (*F*_1,9_ = 11.18, *P* <* *0.01) was found, indicating that NAC significantly decreased active lever responding during extinction compared to vehicle. No session x treatment interaction was observed. Finally, active lever pressing from extinction and reinstatement sessions was analyzed using a two‐way ANOVA. A significant main effect of session (*F*_1,9_ = 36.86, *P* <* *0.0005) was found, indicating that animals pressed the active lever more during reinstatement than during their previous two extinction sessions. A significant main effect of treatment (*F*_1,9_ = 10.54, *P* <* *0.05) was observed, indicating that chronic NAC treatment decreased active lever pressing during extinction and reduced nicotine‐seeking behavior during reinstatement. Furthermore, a significant session x treatment interaction was found (*F*_1,9_ = 8.28, *P* <* *0.05). Bonferroni‐corrected post hoc analyses indicate that treatment with NAC significantly reduced active lever pressing during reinstatement (*P* <* *0.05), but not extinction.

## Discussion

Here, we examined the effects of NAC administration on cue‐induced nicotine reinstatement following withdrawal with extinction training as well as its effects on glutamatergic alterations within the NAcore. Specifically, these studies explored the ability of subchronic NAC treatment to alter NAcore neurobiological alterations associated with nicotine‐seeking behavior. The mechanisms by which NAC upregulates proteins such as GLT‐1 are unknown, thus we examined the ability of NAC to alter mRNA transcript of various glutamatergic targets shown to be involved in drug‐seeking behavior. Contrary to others (e.g., Ramirez‐Niño et al. 2013; Moro et al. 2018), we report that subchronic administration of NAC during extinction from nicotine did not consistently decrease active lever pressing during reinstatement, but important methodological differences distinguish this study from previous investigations, as described in detail below. Furthermore, we report that transcript and protein expression of multiple glutamatergic genes (*Slc1a2*,* Grin2a*,* Grin2b*,* Gria1*, and *Gria2*) were unaffected by subchronic NAC treatment at 100 mg/kg. However, there was a reduction in *Slc1a2* (GLT‐1) transcript expression following NAC administration, which was localized to saline‐yoked animals. We also report no alterations in dendritic morphology following subchronic NAC treatment during the end of extinction. Lastly, we found that chronic administration of NAC (15 days, throughout extinction and prior to reinstatement) significantly decreased extinction responding and nicotine‐seeking during cue‐induced reinstatement.

Withdrawal from drugs of abuse is associated with changes in glutamatergic synapses within the NAcore (Baker et al. 2003; Knackstedt et al. 2009; Moussawi et al. 2011; Gipson et al. 2013a,2013b; Shen et al. 2014; Scofield et al. 2016; Spencer et al. 2017; Kim et al. 2018). As well, NAC is a glutamatergic compound previously shown to reduce reinstated drug‐seeking behavior and reverse or restore altered glutamatergic signaling following various self‐administered drugs of abuse (Moussawi et al. 2009; Kupchik et al. 2012). However, the time course and mechanisms of NACs therapeutic effects are not well understood. In the present study, subchronic administration of a high dose of NAC (100 mg/kg for five sessions) during nicotine withdrawal with extinction training did not consistently decrease active lever pressing during reinstatement (Fig. 1E). Preclinical studies show that NAC inhibits drug‐seeking following an extinction phase where the self‐administered drug has not been available for multiple weeks (Zhou and Kalivas 2008; Ramirez‐Niño et al. 2013; Reissner et al. 2015). Similar to the present extended duration NAC study, some prior studies administered NAC throughout the entire duration of the extinction phase (Zhou and Kalivas 2008; Amen et al. 2011; Reichel et al. 2011). Clinically, NAC has been shown to reduce the self‐reported number of cigarettes smoked per day when administered for 4 weeks (Knackstedt et al. 2009; McClure et al. 2015), however, there was no concurrent reduction in breath carbon monoxide levels (a biomarker of abstinence from smoking) in either study, and most participants did not achieve abstinence. As well, alcohol consumption was a significant covariate in one study (Knackstedt et al. 2009), thus it is unclear if the NAC treatment protocol implemented in that study was sufficient to reverse glutamatergic alterations induced by nicotine/alcohol co‐use. Of note, the NAC administration paradigm utilized in our studies consisted of a between‐subjects design with rats receiving one NAC dose, as opposed to a within‐subjects design (Ramirez‐Niño et al. 2013; Moro et al. 2018). Although subchronic NAC did not decrease nicotine‐seeking, extended duration of NAC treatment at the same dose, beginning on the first day of withdrawal with extinction training, significantly decreased active lever pressing during extinction and attenuated reinstatement of nicotine‐seeking behavior (Fig. 6). Consistent with our results, one study found reductions in cocaine seeking behavior following 12 days of NAC treatment as well as a reduction in lever pressing during extinction with 100 mg/kg NAC compared to vehicle (Reichel et al. 2011). In another study, NAC was administered for 14 days and significantly decreased nicotine self‐administration; an effect that emerged following six continuous injections (Ramirez‐Niño et al. 2013). Taken together, these results suggest that extended NAC treatment is required for consistent reductions in nicotine‐motivated behaviors.

One recent study found that nicotine reinstatement induced by a compound discriminative stimulus (S^D+^)/CS+ was attenuated by one administration of NAC (100 mg/kg; Moro et al. 2018). Contrary to our methodology, Moro et al. (2018) administered a total of three NAC injections, with three washout days between each injection. This treatment protocol reflects acute rather than subchronic or chronic effects of NAC, as only one exposure per NAC dose was given, nonconsecutively. The injection protocol utilized in our studies consisted of consecutive NAC treatment of the same dose across 5 or 15 days prior to and including reinstatement and neurobiological endpoints. An additional methodological difference between our study and Moro et al. (2018) is the duration of nicotine self‐administration (over 30 sessions vs. minimum 10 in our studies). As well, Moro et al. (2018) used a within‐subjects design in which animals received one exposure to each of three conditions: combination S^D+^/CS+, CS+‐alone, or S^D+^‐alone. Importantly, supplemental data in Moro et al. (2018) illustrate contrary results. Although mean active lever pressing was lower in the CS+‐alone condition in NAC‐treated compared to vehicle‐treated animals, cumulative responding in NAC‐treated animals was higher than in vehicle‐treated animals in the CS+‐alone condition as well as in the S^D+^/CS+ condition. Given the discrepancy in reported data within this single study, it is difficult to determine if NAC effectively reduced reinstatement of nicotine‐seeking to different cues, and specifically relevant to our study, to the CS+. Finally, it should be noted that this study did not measure GLT‐1 protein, mRNA, or functional changes after administration of any dose of NAC. Thus, although this study reports that NAC decreased reinstatement at the highest dose tested, Moro et al. (2018) did not confirm that NAcore GLT‐1 was (1) downregulated after nicotine self‐administration in their paradigm or (2) rescued by acute administration of high‐dose NAC.

Here, we propose that the neurobehavioral effects of NAC may be different following nicotine and cocaine use as important differences exist in the primary mechanisms of action as well as use patterns between these two drugs of abuse. For example, nicotine is a full agonist at nicotinic acetylcholine receptors (nAChRs), whereas cocaine blocks the dopamine transporter (DAT). Although both nicotine and cocaine increase dopamine in the NAcore (Di Chiara and Imperato 1988) and NAcore glutamate overflow occurs during cue‐reinstated drug‐seeking following self‐administration of both drugs (Gipson et al. 2013b; Smith et al. 2017), cholinergic and dopaminergic signaling may be altered differentially following chronic nicotine versus cocaine use due to their different mechanisms of action. Specifically, while cocaine acts directly at the DAT, nicotine acts directly at nAChRs located on cholinergic interneurons, glutamatergic afferents, dopaminergic terminals (Mameli‐Engvall et al. 2006), as well as GABAergic interneurons (Exley et al. 2011), each of which can lead to a downstream increase in dopamine (DA) levels. Activation of nAChRs located on cholinergic interneurons results in an increase in release of acetylcholine, as well as excitation of dopaminergic neurons and thus an increase in DA release (Exley and Cragg 2008; Cachope et al. 2012). Nicotinic activation of nAChRs located on glutamatergic afferents increases the firing frequency of dopaminergic cells as well as DA release (Mameli‐Engvall et al. 2006). Furthermore, nicotinic activation of ɑ4‐containing nAChRs located on GABAergic cells within the ventral tegmental area (VTA) can also increase the firing rate of dopaminergic neurons (Exley et al. 2011). Thus, while NAcore dopamine levels are enhanced by both cocaine and nicotine, the mechanisms by which DA levels are modulated are vastly different. Importantly, desensitization of nAChRs by nicotine may enhance the salience of environmental cues associated with smoking behavior (Picciotto et al. 2008), which may underlie the cue dependency of nicotine use (Caggiula et al. 2001). This mechanism may enhance the power of conditioned stimuli to drive motivated nicotine‐seeking during relapse, and may impact the ability of NAC to inhibit craving induced by conditioned cues in smokers. As well, smoking patterns in daily smokers can be characterized by regular frequency of use during the waking day (Chandra et al. 2007). In daily smokers, a consistent number of cigarettes smoked per day across days of the week (~12 cigarettes/day) has been reported, indicating stable chronic use (Shiffman et al. 2014). In preclinical models of nicotine self‐administration, limited access (2 h/day) is typically used, as rodents only escalate nicotine intake with intermittent (every 24–48 h) extended access schedules (Cohen et al. 2012) that, similar to limited access models, do not necessarily mimic human intake patterns. Preclinically, animals readily escalate cocaine intake in models of extended access (Ahmed and Koob 1998). In humans, cocaine use patterns involve intravenous, snorting, or smoking routes of administration, and users commonly report a history of overdose (Pottieger et al. 1992). Given the different patterns of use and mechanisms of action between cocaine and nicotine, different NAC treatment regimens and/or doses may be required to effectively promote smoking cessation.

As noted above, differences between drugs of abuse may impact the ability of NAC to effectively reduce craving and seeking in individuals with different SUDs, and may indicate that different treatment protocols are needed depending on the substance of abuse. While our results are contrary to some previous preclinical and clinical literature (primarily with cocaine: see LaRowe et al. 2006; Reichel et al. 2011; Reissner et al. 2015; although also see LaRowe et al. 2013), they may indicate a timing‐dependent mechanism of NAC as well as a difference in its therapeutic efficacy when used as a pharmacotherapy to treat various SUDs. As well, in LaRowe et al. 2013, only individuals in withdrawal from cocaine reported any beneficial outcomes after NAC administration. Together with our results, clinical and preclinical findings across drugs of abuse suggest that the therapeutic effects of NAC are influenced heavily by the duration of treatment, where NAC may only be clinically therapeutic when given over an extended period of time. Indeed, previous findings across both clinical and preclinical levels of analysis suggest that the therapeutic effects of NAC may only be beneficial for individuals with cocaine use disorder when in a state of withdrawal (Reichel et al. 2011; LaRowe et al. 2013; Reissner et al. 2015). We hypothesize that NAC may not be robustly beneficial for individuals with tobacco use disorder when administered subchronically (i.e. the same treatment protocol as used with cocaine), as only modest reductions in cigarettes smoked per day were reported (see Knackstedt et al. 2009; McClure et al. 2015). However, consistent with the results presented here, chronic NAC treatment given over a period of multiple weeks may promote nicotine abstinence. Interestingly, a relatively robust positive signal with cessation from marijuana use has also been observed in adolescents following 8 weeks of NAC treatment (Gray et al. 2012). However, a recent follow‐up in adult cannabis users found no significant benefits of NAC plus contingency management compared to placebo plus contingency management across a 12‐week study (Gray et al. 2017), though treatment adherence was cited as an underlying issue potentially causing the discrepancy in results between adolescents and adults. Taken together, these studies indicate that long‐term NAC maintenance may be necessary to promote abstinence from some drugs of abuse.

Alterations in GLT‐1 protein expression (Knackstedt et al. 2009, 2010; Reissner et al. 2015) and function (Trantham‐Davidson et al. 2012) have been reported within the nucleus accumbens and several other brain regions (LaRowe et al. 2006) following exposure to drugs of abuse. Antisense knockdown of GLT‐1 prevents subchronic NAC‐induced inhibition of cocaine seeking behavior (Reissner et al. 2015). We have previously found that GLT‐1 protein expression is downregulated after withdrawal with extinction from nicotine self‐administration (at *T* = 0) but rapidly increases at *T* = 15, likely a nicotine cue‐dependent compensatory mechanism in response to glutamate overflow (Gipson et al. 2013a,2013b, 2017). Here we report no difference in GLT‐1 protein expression between vehicle‐treated saline and nicotine animals, however, animals in this experiment received a 2‐h reinstatement session. Thus, it is possible that GLT‐1 protein upregulates rapidly in response to cues and remains elevated throughout a 2‐h reinstatement session. It should be noted that previous studies used membrane subfractionation to approximate changes in GLT‐1 protein trafficking, whereas our study utilized a whole cell lysate preparation (e.g., Gipson et al. 2013a,2013b; Reissner et al. 2015). Furthermore, we report here the novel finding that *Slc1a2* transcript expression is decreased by over 90% in animals reinstated to nicotine conditioned cues compared to saline‐administered animals, indicative of a nearly 13‐fold decrease (Fig. 2D). These results are consistent with the previously reported decrease in NAcore GLT‐1 protein expression following nicotine self‐administration (Knackstedt et al. 2009; Gipson et al. 2013b), though inconsistent with the GLT‐1 protein expression results seen here. The difference in expression levels between mRNA and protein in reinstated animals is potentially indicative of increased translation of GLT‐1 mRNA following extinction or decreased degradation of GLT‐1 protein. As well, others have found that GLT‐1 splice isoform mRNA expression decreases after extended access to and followed by protracted abstinence from cocaine (Kim et al. 2018). However, unlike studies using cocaine that report a NAC‐induced increase in GLT‐1 protein expression (Reissner et al. 2015), we report that GLT‐1 protein was unchanged. Importantly, the intracellular mechanisms by which NAC upregulates GLT‐1 protein are currently unknown. Interestingly, a recent study where GLT‐1a was virally overexpressed following cocaine self‐administration indicated that the increase in GLT‐1 expression reduced glutamate efflux in the NAcore, but did not alter cue‐ or cocaine‐primed reinstatement (Logan et al. 2018), indicating a minimum level of GLT‐1 protein is insufficient to gate cued drug‐seeking. As well, one previous study found that striatal GLT‐1 protein was reduced in a Huntington's disease model, but NAC (500 mg/kg daily for ~9 weeks) was unable to rescue the decrease (Wright et al. 2015). This study is notable because it reported a dissociation between the beneficial effects of chronic exposure to a very high dose of NAC on the diseased state and upregulation of striatal GLT‐1 protein. As well, NAC had no effect on GLT‐1 in wild‐type animals, similar to our yoked saline animals. Akin to beneficial behavioral effects of NAC on drug‐seeking, it is possible that the ability of NAC to restore this nicotine cue‐dependent glutamatergic alteration may only emerge after chronic administration over weeks of withdrawal. Additional studies identifying both short‐ and long‐term effects of NAC treatment on GLT‐1 protein and transcript expression are necessary to confirm these hypotheses.

Previous research has shown that withdrawal from nicotine self‐administration drives NAcore synapses to rest in an enduring state of potentiation, leading to heightened nicotine‐seeking behavior as well as increased extracellular glutamate and dendritic spine potentiation (Gipson et al. 2013b). Furthermore, altered dendritic spine morphology is a conserved neurobiological observation following withdrawal from heroin, Δ^9^‐tetrahydrocannabinol (THC), nicotine, and cocaine (Shen et al. 2011; Dumitriu et al. 2012; Gipson et al. 2013a,2013b; Spencer et al. 2017). Contrary to previous findings, we did not replicate t‐SP at *T* = 15 compared to animals not reinstated to nicotine conditioned cues (*T* = 0; Fig. 4C). Specifically, we show a *decrease* in spine density at *T* = 15, and no change in head diameter or neck length. Although the mechanism for this decrease is unclear, a persistent decrease in NAcore spine density on proximal dendrites was previously found in animals withdrawn from cocaine (enduring up to 28 days; Dumitriu et al. 2012). As well, a recent study found that extinction of THC self‐administration was associated with a persistent decrease in spine density (Spencer et al. 2017). One important difference between the current study and previous publications is that the analysis was pooled by animal, and not by number of spines. Although it has previously been shown that reinstated cocaine seeking behavior is positively correlated with magnitude of t‐SP (Gipson et al. 2013a), this was not reported in nicotine‐seeking animals (Gipson et al. 2013b). In both studies, both behavior and spine head diameter was increased simultaneously after 15 min of reinstatement. Here we show a previously unreported dissociation between spine head diameter change and motivated drug‐seeking behavior. This is of particular importance as we begin to uncover the relevance of structural changes in plasticity for motivated behaviors, as well as the relevance of t‐SP as a biomarker for relapse treatment (Gipson and Olive 2017).

A novel finding in the current studies is that NAC treatment did not significantly affect mean spine head diameter during initiated cued nicotine‐seeking (Fig. 5). Additionally, neither cumulative frequency distributions of spine head diameter nor mean spine density between treatment groups yielded significant differences. One potential limitation of the current study is that animals in the 100 mg/kg NAC spine group displayed significantly more nicotine intake than vehicle‐treated animals (Fig. 5C). However, a correlational analysis between nicotine infusions and mean spine head diameter revealed no correlation or significant non‐zero slope, indicating nicotine intake prior to extinction and reinstatement has no significant effect on dendritic spine head diameter. Our results suggest that subchronic NAC treatment does not differentially impact spines based on their morphology in a behavior‐dependent manner (Arellano 2007; De Roo et al. 2008). As well, NAC did not restore the decrease in spine density at *T* = 15, consistent with the lack of nicotine reinstatement inhibition. Although previous studies have equated an increase in spine density with synaptic potentiation (Dumitriu et al. 2010), others have shown either no change in spine density but dynamic changes in spine head diameter following self‐administration of nicotine and cocaine (Gipson et al. 2013a,2013b), or a decrease in spine density following THC self‐administration (Spencer et al. 2017). Interestingly, the reduction in spine density seen here has previously been observed with opiates but not stimulants (see review by Russo et al. 2010), though both classes of drugs produce addictive phenotypes. This potentially indicates that the reduction in density during initiated reinstatement seen here represents an increase in synaptic efficacy comparable to that caused by an increase in other t‐SP features, including increased spine head diameter. Consistent with NAC's inability to inhibit nicotine‐seeking behavior (Figs. 1E, 2C, 3C, and 5D), results at *T* = 15 indicate no effect of NAC on spine head diameter, and an inability to restore the decrease in spine density (Fig. 5E and G). However, it is unclear if NAC inhibits functional t‐SP measured via changes in AMPA currents (Gipson et al. 2013a). Although the dose used here is relatively high (100 mg/kg) and has been used in preclinical behavioral models of drug relapse, it may not be high enough to reverse all nicotine‐induced neurobiological changes associated with relapse vulnerability. Contrary to previous findings that NAcore t‐SP may be a neurobiological consequence of drug‐seeking behavior (Gipson et al. 2013a,2013b; Stefanik et al. 2016), the results seen here indicate no significant potentiation of NAcore spines either when collapsing across number of animals or in the cumulative frequency distributions of spine head diameters from *T* = 0 and *T* = 15 animals (Fig. 4C and D, respectively).

Spine neck length is an LTP‐related measurement (Yuste & Bonhoeffer 2001), and recent work has indicated an increase in efficacy of synapses as a function of neck length. For example, two‐photon uncaging of glutamate near spine heads of mouse pyramidal neurons demonstrated that the amplitude of the uncaged potentials at the soma was inverse to spine neck length, independent of spine position or spine head size (Araya et al. 2006). Furthermore, in longer spine necks activated by uncaged glutamate, calcium transients had no discernible impact on somatic voltage depolarization, indicating that synapses can be electrically isolated by increasing neck length (Araya et al. 2006). In contrast, activation of a postsynaptic spike simultaneous to uncaging glutamate causes rapid decreases in spine neck length with increased evoked potentials seen at the soma (Araya et al. 2014). Spine neck geometry also regulates spine calcium signaling, with neck length and neck radius regulating conductance between spine head and dendrite (Noguchi et al. 2005). Thus, it is hypothesized that spine neck shortening acts as a plasticity mechanism to increase synaptic efficacy, engaging the spine with dendrite and soma (Araya et al. 2014). Here we report no alterations in spine neck length distribution in animals that receive contingent cue reinstatement (Fig. 4F). As well, no change in spine neck length was found following administration of NAC (Fig. 5H). Although it is possible that the shortening of the spine neck is an important neurobiological event associated with LTP, changes in spine neck length may not be a relevant neurobiological phenomenon during nicotine relapse.

## Conclusions and Future Directions

Tobacco smoking remains one of the leading causes of avoidable death in the US (Xu 2013). Currently available treatments for tobacco use disorder have inconsistent clinical success in promoting long‐term smoking cessation (Cahill et al. 2014) and often are associated with adverse side effects. Thus, novel therapeutic options are greatly needed. NAC appears to be a promising, well‐tolerated pharmacotherapy in the treatment of tobacco and other SUDs. However, our data and others indicate that treatment regimen should be carefully tailored, including dose, route, and duration of administration, and dependent upon whether the individual is in withdrawal. Our results aide in the current understanding of NAC as a treatment for SUDs, as our findings indicate that duration of NAC treatment is a critical factor to its therapeutic efficacy.

## Conflict of Interest

The authors declare no conflict of interest.
